# Eribulin versus dacarbazine in patients with leiomyosarcoma: subgroup analysis from a phase 3, open-label, randomised study

**DOI:** 10.1038/s41416-019-0462-1

**Published:** 2019-05-08

**Authors:** Jean-Yves Blay, Patrick Schöffski, Sebastian Bauer, Anders Krarup-Hansen, Charlotte Benson, David R. D’Adamo, Yan Jia, Robert G. Maki

**Affiliations:** 1 Department of Medical Oncology, Université Claude Bernard & Centre Léon Bérard, Lyon, France; 2 Department of General Medical Oncology, University Hospitals Leuven, Leuven, Belgium; 3 Department of Medical Oncology, Sarcoma Center, West German Cancer Center, University of Duisburg-Essen, Essen, Germany; 4 Department of Oncology, Herlev Hospital-University of Copenhagen, Herlev, Denmark; 5 Sarcoma Unit, Royal Marsden NHS Foundation Trust, London, UK; 6 Eisai Inc, Woodcliff Lake, NJ USA; 7 Northwell Health Cancer Institute, Monter Cancer Center, Lake Success, NY USA; 8 Cold Spring Harbor Laboratory Cancer Center, Cold Spring Harbor, NY USA

**Keywords:** Sarcoma, Sarcoma, Sarcoma, Sarcoma

## Abstract

**Background:**

This subgroup analysis of a phase 3 study compares outcomes for eribulin versus dacarbazine in patients with leiomyosarcoma.

**Methods:**

Patients ≥18 years old with advanced liposarcoma or leiomyosarcoma, ECOG PS ≤2, and ≥2 prior treatment regimens were randomly assigned (1:1) to eribulin mesylate (1.4 mg/m² intravenously on day 1 and day 8) or dacarbazine (either 850, 1000, or 1200 mg/m² intravenously) every 21 days until disease progression. The primary end point was OS; additional end points were progression-free survival (PFS) and objective response rate (ORR).

**Results:**

309 Patients with leiomyosarcoma were included (eribulin, *n* = 157; dacarbazine, *n* = 152). Median age was 57 years; 42% of patients had uterine disease and 57% had nonuterine disease. Median OS was 12.7 versus 13.0 months for eribulin versus dacarbazine, respectively (hazard ratio [HR] = 0.93 [95% CI 0.71–1.20]; *P* = 0.57). Median PFS (2.2 vs 2.6 months, HR = 1.07 [95% CI 0.84–1.38]; *P* = 0.58) and ORR (5% vs 7%) were similar between eribulin- and dacarbazine-treated patients. Grade ≥3 TEAEs occurred in 69% of patients receiving eribulin and 59% of patients receiving dacarbazine.

**Conclusions:**

Efficacy of eribulin in patients with leiomyosarcoma was comparable to that of dacarbazine. Both agents had manageable safety profiles.

## Background

Soft tissue sarcomas (STSs) consist of a heterogeneous group of mesenchymal tumours and comprise numerous histologically distinct subtypes that affect various primary sites. As a group, STS types are rare, with an incidence rate of 3–5 cases per 100,000 persons per year.^1,2^ Liposarcoma (LPS) and leiomyosarcoma (LMS) are two of the more frequently observed sarcoma subtypes, both of which are further categorised based on distinctive clinical and pathologic features.^2–4^ LMS represents ~20% of STS cases and forms in smooth muscle, most often in the branches of large veins such as the inferior vena cava, or in the uterus.^2,4^

Patients with metastatic STS have a poor prognosis, with disease-specific survival rates at 5 years of ~15%.^5^ For unresectable or metastatic disease, anthracycline-based chemotherapy is the recommended first-line treatment, with variations in practice between use of doxorubicin as a single agent or in combination with other agents (typically with olaratumab, ifosfamide, or dacarbazine).^6–8^ Dacarbazine has shown single-agent activity and improved efficacy in combination with doxorubicin in patients with LMS.^9^ In a randomised phase 1b/2 trial in anthracycline-naive patients with advanced STS, the combination of doxorubicin and olaratumab significantly improved overall survival (OS) compared with doxorubicin alone. Results of the confirmatory phase 3 trial are pending.^10^

Several treatment options, including ifosfamide, trabectedin, and pazopanib, are available for second- or later-line therapy for STS (excluding LPS).^11–14^ Although not approved for sarcoma, gemcitabine, alone or in combination with either docetaxel or dacarbazine, has demonstrated activity as second-line therapy in STS and is frequently used in clinical practice.^15,16^ In recent years, subtype-specific sensitivity to drugs has become more apparent and several trials have focused on patient populations with specific STS subtypes. Differing sensitivity between sarcoma subtypes has been observed with other agents, for example, myxoid round cell LPS is particularly sensitive to treatment with trabectedin.^11^ Recent data suggest that there are different molecular subtypes in LMS and that these subtypes are associated with distinct clinical outcomes.^17^ This finding suggests potential treatment of LMS using a more targeted approach.

Eribulin was approved in 2016 in the United States and European Union for treatment of unresectable or metastatic LPS in patients whose disease had failed to respond to anthracycline chemotherapy.^18,19^

Eribulin is a structurally modified, synthetic analogue of halichondrin B, a natural product isolated from the marine sponge *Halichondria okadai*.^20^ It is a nontaxane inhibitor of microtubule dynamics, with a unique mechanism of action compared with vinca alkaloids.^20–22^ In preclinical studies, eribulin affected tumour biology, including vascular remodelling, reversal of the epithelial-to-mesenchymal transition, induction of differentiation, and suppression of migration and invasion.^23–25^

In a phase 2 study of eribulin in STS, 32% of patients with LMS and 47% of patients with LPS met the primary end point of being progression free at 12 weeks.^26^ Based on these results, eribulin was evaluated in an international phase 3 study of patients with unresectable or metastatic advanced LPS or LMS, in which eribulin significantly improved OS compared with dacarbazine in the total study population (13.5 vs 11.5 months, hazard ratio [HR] 0.77 [0.62–0.95], *P* = 0.017).^18^ Here, we present the results of a histology-driven analysis of the efficacy and safety of eribulin in patients with LMS, a randomised and stratified subgroup enrolled in the phase 3 study.

## Methods

### Study design and participants

The design of the phase 3, randomised, open-label study (NCT01327885) evaluating the effects of eribulin compared to dacarbazine in patients with advanced LPS or LMS has been previously published.^18^ In brief, patients were randomly assigned (1:1) between March 2011 and May 2013 to treatment groups and stratified by the diagnosis of LPS or LMS, geographic region, and the number of previous chemotherapy regimens. Eligible patients were 18 years or older and had histologically confirmed LPS or LMS with ≥2 prior chemotherapy treatments for advanced disease. Entry criteria also required radiographic evidence of disease progression within 6 months prior to randomisation and the presence of measurable disease according to Response Evaluation Criteria In Solid Tumours version 1.1 (RECIST v1.1).^27^ The study was conducted at 110 sites in 22 countries under the principles of good clinical practice and the World Medical Association’s Declaration of Helsinki (2008), and was approved by the respective institutional research ethics boards of each participating site (see Supplementary Table 1). All patients provided written informed consent.

### Treatment

Eribulin mesylate was given at a dose of 1.4 mg/m^2^ intravenously (equivalent to 1.23 mg/m^2^ eribulin [expressed as free base]) on day 1 and day 8 of each 21-day cycle. Dacarbazine was given at a dose of either 850, 1000, or 1200 mg/m^2^ intravenously on the first day of every 21-day cycle. The dacarbazine starting dose was selected by the investigator prior to randomisation based on each patient’s clinical status and local institutional guidelines. Treatment continued until disease progression, unacceptable toxicity, or withdrawal of consent.

Tumours were assessed using computed tomography scans or magnetic resonance imaging every 6 weeks for the first 12 weeks and then every 9 weeks thereafter until disease progression. Tumour responses and progression status were determined based on investigator review, following RECIST v1.1, except that chest lesions could not be assessed using X-ray. Safety was assessed by monitoring for treatment-emergent adverse events (TEAEs) and regular evaluation of clinical laboratory test results, vital signs, electrocardiography scans, and physical examinations.

### Statistical analysis

The primary end point was OS, and secondary end points included progression-free survival (PFS), progression-free rate at 12 weeks, clinical benefit rate (defined as the proportion of patients whose best overall response was complete response [CR], partial response [PR], or durable stable disease [defined as the proportion of patients who experienced SD for ≥11 weeks]), pharmacokinetics, and safety. Exploratory end points included objective response rate (ORR; defined as the proportion of patients whose best overall response was either CR or PR), and disease control rate (defined as the proportion of patients whose best overall response was CR, PR, or SD). Subgroup analysis based on histologic diagnosis, along with other baseline demographic and disease factors, was prespecified in the study protocol. This study was neither designed nor powered to draw definitive conclusions on the activity of eribulin in histologic subgroups.

OS and PFS were estimated with the Kaplan–Meier product-limit method, and 95% confidence intervals (CIs) were constructed using Greenwood’s formula. HRs were based on a Cox regression model, including treatment as a covariate and stratification factors of geographic region and number of prior chemotherapy regimens. *P* values were calculated using a two-sided stratified log-rank test with the same stratification factors as for HRs. Efficacy analyses were performed in the intent-to-treat population, comprising all patients who were randomly assigned to treatment. Safety data were summarised descriptively based on all randomised patients who had received at least 1 dose of study treatment and had at least 1 posttreatment safety evaluation.

## Results

### Patients

This is a histology-driven subgroup analysis from a large, prospective, randomised, phase 3 trial of eribulin in which 452 patients with advanced LPS or LMS in the intent-to-treat population (Supplementary Fig. 1) were randomly assigned to receive eribulin (*n* = 228) or dacarbazine (*n* = 224). A total of 309 patients with LMS were included in this analysis; 157 patients were treated with eribulin and 152 were treated with dacarbazine (Table 1). Patients with LMS composed 68% of the overall patient population in the phase 3 study. Within the LMS subgroup, 131 (42%) patients had uterine disease and 177 (57%) patients had nonuterine disease (Table 1). Baseline demographic and disease characteristics were generally well balanced between treatment arms.

**Table 1:** Demographic, baseline, and disease characteristics of patients with leiomyosarcoma

Characteristic	Eribulin (*n* = 157)	Dacarbazine (*n* = 152)	Total (*N* = 309)
Median age (minimum, maximum), years	57 (28, 76)	56 (24, 77)	57 (24, 77)
Age group, *n* (%), years			
<65	123 (78)	124 (82)	247 (80)
≥65	34 (22)	28 (18)	62 (20)
Sex, *n* (%)			
Male	29 (18)	31 (20)	60 (19)
Female	128 (82)	121 (80)	249 (81)
Race, *n* (%)			
White	110 (70)	117 (77)	227 (74)
African American	6 (4)	4 (3)	10 (3)
Asian^a^	14 (9)	12 (8)	26 (8)
Other^b^	27 (17)	19 (12)	46 (15)
ECOG PS, *n* (%)			
0	76 (48)	66 (43)	142 (46)
1	80 (51)	79 (52)	159 (52)
2	1 (1)	7 (5)	8 (3)
Histology subcategory, *n* (%)			
Uterine	68 (43)	63 (41)	131 (42)
Nonuterine	88 (56)	89 (59)	177 (57)
Tumour grade, *n* (%)			
High	112 (71)	113 (74)	225 (73)
Intermediate	45 (29)	37 (24)	82 (27)
Not done	0	2 (1)	2 (1)
Geographic region, *n* (%)			
USA and Canada	62 (40)	61 (40)	123 (40)
Western Europe, Australasia, Israel	70 (45)	68 (45)	138 (45)
Eastern Europe, Latin America, Asia	25 (16)	23 (15)	48 (16)
Median age at diagnosis (minimum, maximum), years	53.0 (24, 75)	52.5 (23, 75)	53.0 (23, 75)
Previous anticancer therapy,^c^ *n* (%)			
0	0	0	0
1	2 (1)	1 (1)	3 (1)
2	76 (48)	70 (46)	146 (47)
3	44 (28)	44 (29)	88 (28)
4	17 (11)	25 (16)	42 (14)
>4	18 (12)	12 (8)	30 (10)

*ECOG PS* Eastern Cooperative Oncology Group performance status

^a^Includes Japanese, Chinese, and other Asian

^b^Includes American Indian or Alaskan Native, Native Hawaiian or Other Pacific Islander, other, and not applicable

^c^Excludes radiotherapy and surgery

### Efficacy

**Fig. 1:**
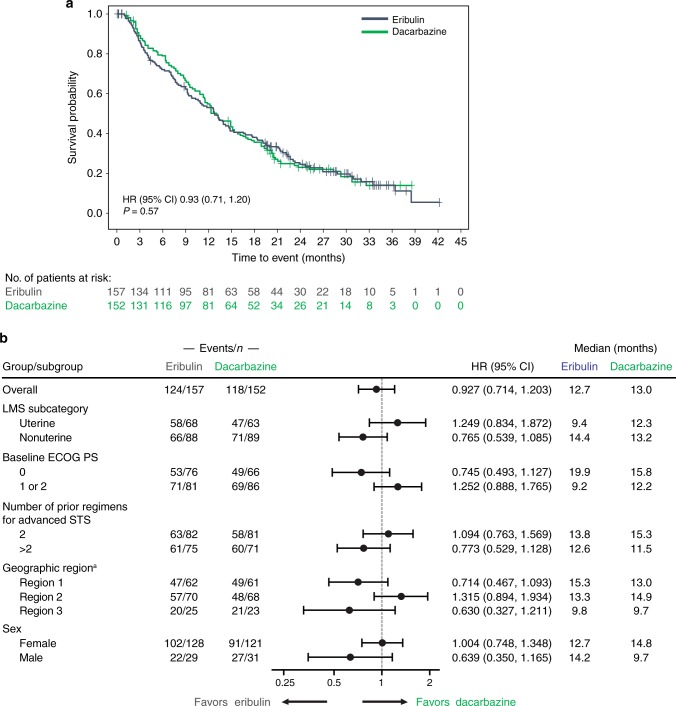
Kaplan–Meier curve of overall survival (**a**) and hazard ratios of overall survival (**b**) in patients with leiomyosarcoma. CI confidence interval, ECOG PS Eastern Cooperative Oncology Group performance score, HR hazard ratio (eribulin to dacarbazine), based on a Cox regression model including treatment as a covariate and stratification factors of geographic region and number of prior chemotherapy regimens, LMS leiomyosarcoma, STS soft tissue sarcoma. ^a^ Region 1 = USA and Canada; Region 2 = Western Europe, Australasia, and Israel; Region 3 = Eastern Europe, Latin America, and Asia

In patients with LMS, the median OS was 12.7 vs 13.0 months for eribulin and dacarbazine, respectively (HR = 0.93 [95% CI 0.71–1.20]; *P* = 0.57) (Fig. 1a, b). HRs for OS favoured eribulin treatment, but were not statistically different from dacarbazine treatment, in patients with LMS who had >2 prior chemotherapy treatments for advanced disease, had nonuterine disease, had a baseline Eastern Cooperative Oncology Group performance status of 0, were enrolled in geographic region 1 (North America) or geographic region 3 (Eastern Europe, Latin America, and Asia), or were male (Fig. 1b). Median OS for patients with uterine LMS was 9.4 versus 12.3 months for eribulin and dacarbazine, respectively, which was not statistically significantly different (HR = 1.25 [95% CI 0.83–1.87]) (Fig. 1b, Supplementary Fig. 2a). For patients with nonuterine disease, median OS was 14.4 versus 13.2 months for eribulin and dacarbazine, respectively, again not statistically significantly different (HR = 0.77 [95% CI 0.54–1.09]) (Fig. 1b, Supplementary Fig. 2a). Male patients treated with eribulin had numerically longer OS compared with male patients taking dacarbazine (14.2 vs 9.7 months, respectively) (Fig. 1b, Supplementary Fig. 3a), but this difference was not statistically significant.

**Fig. 2:**
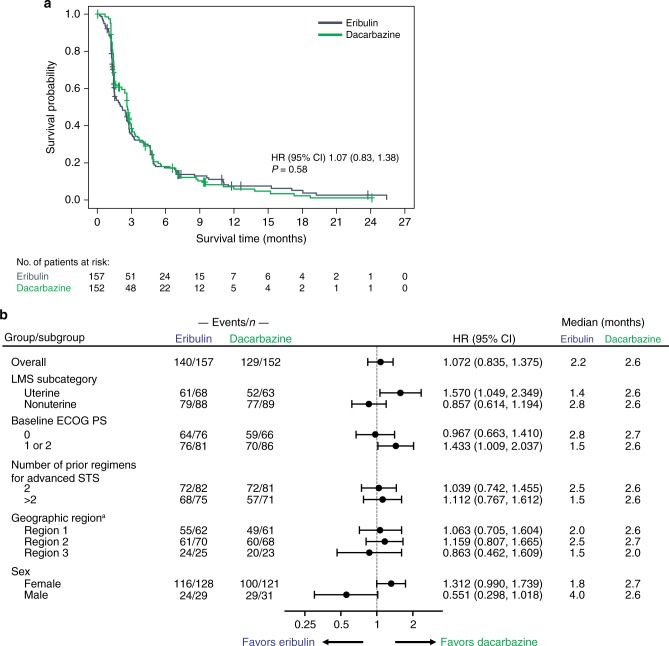
Kaplan–Meier curve of progression-free survival (**a**) and hazard ratios of progression-free survival (**b**) in patients with leiomyosarcoma. CI confidence interval, ECOG PS Eastern Cooperative Oncology Group performance score, HR hazard ratio (eribulin to dacarbazine), based on a Cox regression model including treatment as a covariate and stratification factors of geographic region and number of prior chemotherapy regimens, LMS leiomyosarcoma, STS soft tissue sarcoma. ^a^ Region 1 = USA and Canada; Region 2 = Western Europe, Australasia, and Israel; Region 3 = Eastern Europe, Latin America, and Asia

Median PFS in patients with LMS was similar between treatment arms (2.2 vs 2.6 months with eribulin and dacarbazine, respectively, HR = 1.07 [95% CI 0.84–1.38]; *P* = 0.58) (Fig. 2a, b). In patients with uterine LMS, median PFS was 1.4 vs 2.6 months for eribulin and dacarbazine, respectively (HR = 1.57 [95% CI 1.05–2.35]) (Fig. 2b, Supplementary Fig. 2b). In patients with nonuterine LMS, median PFS was 2.8 vs 2.6 months for eribulin and dacarbazine, respectively; this difference was not statistically significant (HR = 0.86 [95% CI 0.61–1.19]) (Fig. 2b, Supplementary Fig. 2b). Male patients treated with eribulin had numerically (but not statistically significantly) longer PFS compared with male patients taking dacarbazine (4.0 vs 2.6 months, respectively) (Fig. 2b, Supplementary Fig. 3b).

ORR was similar between eribulin-treated and dacarbazine-treated patients (5% vs 7% respectively). The exploratory tumour response end points, disease control rate and durable stable disease rate (defined as the proportion of patients with SD ≥11 weeks) were numerically higher in patients treated with dacarbazine than in those receiving eribulin (57% [95% CI 48–64%] vs 52% [95% CI 44–60%] and 45% [95% CI 37–54%] vs 36% [95% CI 28–44%], respectively) (Table 2); these differences were not statistically significant.

**Table 2:** Summary of tumour responses by investigator assessment

Parameter	Eribulin (*n* = 157)	Dacarbazine (*n* = 152)
Best overall response		
CR, *n* (%)	0	0
PR, *n* (%)	8 (5)	11 (7)
SD, *n* (%)	73 (47)	75 (49)
PD, *n* (%)	69 (44)	56 (37)
Not evaluable, *n* (%)	2 (1)	1 (1)
Unknown, *n* (%)	5 (3)	9 (6)
Objective response rate		
ORR (95% CI)	5 (2, 10)	7 (4, 13)
Disease control rate		
DCR (95% CI)	52 (44, 60)	57 (48, 64)
Durable stable disease rate		
dSD (95% CI)	36 (28, 44)	45 (37, 54)

*CI* confidence interval, *CR* complete response, *DCR* disease control rate (defined as proportion of PR+CR+SD), *dSD* durable stable disease (defined as the proportion with stable disease for ≥11 weeks), *HR* hazard ratio, *ORR* objective response rate (defined as the proportion of CR + PR), *PFS* progression-free survival, *PD* progressive disease, *PR* partial response, *SD* stable disease

### Safety

In patients with LMS, the five most frequent TEAEs with eribulin were neutropenia (46%), fatigue (46%), nausea (41%), alopecia (33%), and constipation (33%). The five most frequent TEAEs with dacarbazine were nausea (49%), fatigue (41%), thrombocytopenia (31%), anaemia (29%), and constipation (27%) (Table 3). Grade ≥3 TEAEs were reported in 69% of patients in the eribulin arm versus 59% of patients in the dacarbazine arm. Grade ≥3 neutropenia and leukopenia occurred more frequently in patients treated with eribulin, whereas grade ≥3 anaemia and thrombocytopenia occurred more frequently in patients treated with dacarbazine (Table 3).

**Table 3:** Treatment-emergent adverse events ≥10% (all grades, either arm) in patients with leiomyosarcoma

TEAE category, *n* (%)	Eribulin (*n* = 156)			Dacarbazine (*n* = 152)		
	All grades	Grade 3	Grade 4	All grades	Grade 3	Grade 4
Neutropenia	72 (46)	36 (23)	25 (16)	40 (26)	16 (11)	8 (5)
Fatigue	71 (46)	6 (4)	0	63 (41)	3 (2)	0
Nausea	64 (41)	1 (1)	0	74 (49)	1 (1)	0
Alopecia	51 (33)	1 (1)	0	5 (3)	0	0
Constipation	51 (33)	2 (1)	0	41 (27)	1 (1)	0
Anaemia	49 (31)	10 (6)	2 (1)	44 (29)	15 (10)	4 (3)
Pyrexia	46 (30)	2 (1)	0	20 (13)	0	0
Asthenia	35 (22)	2 (1)	0	34 (22)	4 (3)	0
Cough	34 (22)	0	0	24 (16)	0	0
Headache	32 (21)	0	0	17 (11)	0	0
Peripheral sensory neuropathy	30 (19)	2 (1)	0	6 (4)	0	0
Vomiting	30 (19)	2 (1)	0	34 (22)	0	0
Abdominal pain	28 (18)	3 (2)	1 (1)	21 (14)	6 (4)	0
Decreased appetite	27 (17)	1 (1)	0	21 (14)	0	0
Dyspnoea	27 (17)	3 (2)	1 (1)	25 (16)	3 (2)	2 (1)
Back pain	26 (17)	2 (1)	0	23 (15)	3 (2)	0
Diarrhoea	25 (16)	0	0	22 (15)	0	0
Leukopenia	25 (16)	12 (8)	4 (3)	20 (13)	5 (3)	3 (2)
Stomatitis	22 (14)	2 (1)	0	4 (3)	1 (1)	0
Oedema peripheral	21 (14)	0	0	11 (7)	1 (1)	0
Abdominal pain, upper	17 (11)	0	0	8 (5)	1 (1)	0
Hypokalemia	17 (11)	4 (3)	0	7 (5)	2 (1)	0
Aspartate aminotransferase increased	16 (10)	1 (1)	0	5 (3)	2 (1)	0
Myalgia	16 (10)	0	0	16 (11)	0	0
Urinary tract infection	16 (10)	2 (1)	0	10 (7)	0	0
Thrombocytopenia	10 (6)	1 (1)	0	47 (31)	13 (9)	13 (9)

*TEAE* treatment-emergent adverse event

Nonfatal serious adverse events occurred in 33% of patients in the eribulin arm and 32% of patients in the dacarbazine arm (Supplementary Table 2). TEAEs leading to treatment discontinuation occurred in 8% of patients in the eribulin arm and 5% of patients in the dacarbazine arm, and 16% of patients in the dacarbazine arm required a dose reduction because of TEAEs compared with 28% of patients in the eribulin arm (Supplementary Table 2). However, the frequency of dose interruptions was similar between eribulin- and dacarbazine-treated patients (34% each). A total of 10 patients in the LMS subgroup died from TEAEs, including seven patients in the eribulin arm and three patients in the dacarbazine arm (Supplementary Table 2). In the eribulin arm, only a single event—neutropenic sepsis—was considered possibly related to treatment. None of the three deaths in the dacarbazine arm were considered by the investigators to be treatment related.

## Discussion

We present the results from a histology-driven subgroup analysis of patients with LMS from a large-scale, prospective, randomised, controlled, phase 3 study comparing the efficacy and safety of eribulin to dacarbazine in previously treated patients with advanced STS of two histologically distinct types: LMS and LPS.^18^ The study design was randomised and stratified based on these histological subtypes; however, the study was neither designed nor powered to draw definitive conclusions on the activity of eribulin in histologic subgroups. The phase 3 study was also not designed to be a noninferiority trial.

In this subgroup analysis of patients with LMS, median OS was comparable between patients treated with eribulin versus dacarbazine (HR = 0.93; 95% CI [0.71–1.20]). Secondary outcomes of PFS (HR = 1.07; 95% CI [0.84–1.38]) and ORR (5% vs 7%, respectively) were also comparable between eribulin and dacarbazine treatment groups. Historical data suggest that dacarbazine is more active in LMS than in other sarcomas. In a randomised trial of doxorubicin every 3 weeks versus weekly doxorubicin versus doxorubicin plus dacarbazine for metastatic STS, the combination demonstrated a significant improvement in response rates in patients with LMS compared with doxorubicin alone (44% vs 17% [weekly] vs 20% [every 3 weeks], respectively).^9^ That trial included 99 (36%) of 275 evaluable patients with LMS. By comparison, this phase 3 trial enrolled >300 (309/452; 68%) patients with LMS.

This subgroup analysis supports the hypothesis that treatment outcomes for a particular sarcoma histology will differ as a function of its primary anatomic site.^2,28^ In patients with nonuterine LMS, HRs for OS and PFS favoured eribulin, whereas in patients with uterine LMS, HRs for OS and PFS favoured dacarbazine. However, further research is required to determine if various primary LMS sites have molecular differences that would suggest differential antitumor activity of eribulin between uterine and nonuterine LMS and between male and female patients. Emerging data suggest that there are differences in molecular profiles between uterine and nonuterine LMS.^17^ Differing sensitivity between sarcoma subtypes has been seen with other agents, for example, myxoid round cell LPS is particularly sensitive to treatment with trabectedin, whereas all LPS subtypes are equally sensitive to doxorubicin.^11^ These results may highlight a need to refine stratification criteria in STS trials to include not only disease histology, but also disease site and molecular biology.

Dacarbazine and eribulin each had different TEAE profiles but, overall, the toxicity profiles of both chemotherapy agents in patients with LMS were manageable. Adverse events did not often lead to study-drug discontinuation. No new safety signals were identified, and adverse events associated with eribulin were similar to previous findings.^26^

In summary, this subgroup analysis suggests that the effects of eribulin treatment in patients with LMS are comparable to those of dacarbazine. This contrasts with the results of a subgroup analysis of patients with LPS, in which an OS benefit of eribulin vs dacarbazine was evident.^29^ Outcome heterogeneity within the LMS subgroup may represent biologic differences in this sarcoma subtype. To further understand unique sarcoma subtypes, future prospective trials in STS should aim to examine treatment outcomes as a function of histology, molecular alteration, anatomic site, and gender. The LMS subtype encompasses different disease types based on clinical and pathologic features, and future research should benefit from additional stratification criteria. Such research may contribute to further improvement in outcomes for patients with LMS.

## Supplementary information

Supplementary Table 1

Supplementary Table 2

Supplementary Figures

## Data Availability

The data of this study are considered commercially proprietary and are not stored for unrestricted access. All the authors had full access to all the data in the study and take responsibility for the integrity of the data and the accuracy of the data analysis.
