# Supplementary material for: Eribulin versus dacarbazine in patients with leiomyosarcoma: subgroup analysis from a phase 3, open-label, randomised study
Source: Br J Cancer. 2019 May 8;120(11):1026–32. doi: 10.1038/s41416-019-0462-1 (PMC6738064; doi:10.1038/s41416-019-0462-1)
Supplement: Supplementary file 1 — Supplementary Table 1 [file 41416_2019_462_MOESM1_ESM.docx]

**Supplementary Table 1**

| Quorum Review IRB, USA |
| --- |
| Rush University Medical Center Institutional Review Board, USA |
| Northwestern University Office for the Protection of Research Subjects Institutional Review Board, USA |
| Western Institutional Review Board, USA |
| Office for Human Research Studies, USA |
| University of Southern California Health Sciences Campus Institutional Review Board, USA |
| Washington University in St. Louis School of Medicine Human Research Protection Office, USA |
| MD Anderson Cancer Center Institutional Review Board, USA |
| University of Chicago Institutional Review Board, USA |
| Cedars-Sinai Medical Center Institutional Review Board, USA |
| Office of the Human Research Protection Program, USA |
| Cleveland Clinic Institutional Review Board, USA |
| Kootenai Medical Center IRB, USA |
| Duke University Health System IRB, USA |
| OHSU Institutional Review Board, USA |
| Christiana Care Institutional Review Board, USA |
| Medstar Health Research Institute – Georgetown University Oncology Institutional Review Board, USA |
| University of Pennsylvania Office of Regulatory Affairs, USA |
| Institutional Review Board Roswell Park Cancer Institute, USA |
| Biomedical Research Alliance of New York (BRANY), USA |
| Stanford University Administrative Panel on Human Subjects in Medical Research, USA |
| McGill Faculty of Medicine – Institutional Review Board, Canada |
| Research Ethics Board Mount Sinai Hospital, Canada |
| The Ottawa Hospital Research Ethics Board, Canada |
| The Metro South Hospital and Health Service District Human Research Ethics Committee, Australia |
| Sydney Local Health District Ethics Review Committee, Australia |
| Ethikkommission der Medizinischen Universität Wien und des AKH der Stadt Wien Borschkegasse, Austria |
| Ethikkommission der Medizinischen Universität Graz, Austria |
| Commissie Medische Ethiek – Toetsingscommissie UZ Leuven, Belgium |
| Association Hospitalière de Bruxelles Centre des Tumeurs de l'ULB Comité Ethique, Belgium |
| Comité d'éthique Hospitalo Facultaire Universitaire de Liège, Belgium |
| De Videnskabsetiske Komiteer for Region Hovedstaden, Denmark |
| Comite de Protection des Personnes Ouest II Maison de la Recherche Clinique CHU, France |
| Ethikkommission der Technischen Universitat Dresden, France |
| Medizinische Ethikkommission II Medizinische Fakultät Mannheim der Universität Heidelberg, Germany |
| Ethik-Kommission der MHH, OE 9515, Hannover, Germany |
| Ethik-Kommission der Medizinischen Fakultät der Universität Duisburg-Essen, Germany |
| Ethikkommission der Medizinischen Fakultat und am Universitiitsklinikum Tijbingen, Germany |
| Landesamt fur Gesundheit und Soziales Ethikkommission des Landes Berlin, Germany |
| Ethikkommission Universität zu Köln, Germany |
| Comitato Etico Instituto Oncologico Veneto I.R.C.C.S., Padova, Italy |
| Comitato Etico Independente Fondazione IRCCS., Istituto Nazionale dei Tumori, Milano,Italy |
| Comitato Etico IRCCS lstituto Europeo di Oncologia, Milano, Italy |
| Comitato Etico IRCCS Fondazione Piemonte per l'Oncologia, Candiolo, Italy |
| Comitato Etico Istituto Clinico Humanitas IRCCS, Milano, Italy |
| Comitato Etico Interaziendale A.O.U. San Luigi Gonzaga di Orbassano, Italy |
| Comitato Etico Indipendente Centro di Riferimento Oncologico (CRO), Aviano, Italy |
| Comitato Etico Scientifico Azienda Ospedaliera Ospedale Niguarda, Milano, Italy |
| Comitato Etico della Provincia di Monza e Brianza, Italy |
| Academic Medical Center Medical Ethics Committee, Amsterdam, The Netherlands |
| Comite Etico de Investigacion Clinica Hospital Universitari Germans Trias i Pujol, Barcelona, Spain |
| NRES Committee North West – Greater Manchester Central, UK |
| Institutional Review Board of the Faculty of Medicine, Chulalongkorn University, Bangkok, Thailand |
| Research Ethic Committee, Faculty of Medicine, Chiang Mai University, Thailand |
| Office of Human Research Ethics Committee, Faculty of Medicine, Prince of Songkia University, Thailand |
| Singhealth Centralized IRB, Singapore |
| Severance Hospital Yonsei University Health System Institutional Review Board, Seoul, Republic of Korea |
| Samsung Medical Center Institutional Review Board, Seoul, Republic of Korea |
| Korean University Anam Hospital Institutional Review Board, Seoul, Republic of Korea |
| Seoul National University Bundang Hospital, Institutional Review Board, Republic of Korea |
| Eticka Komise FN Hradec Kralove, Czech Republic |
| Eticka Komise Fakultni nemocnice v Motole, Czech Republic |
| Eticka Komise Masarykuv Onkologicky ustav Brno, Czech Republic |
| Eticka Komise pri Nemocnici Novy Jicin, Czech Republic |
| Komisja Bioetyczna przy Centrum Onkologii Instytucie im. Marii Sklodowskiej – Curie w Warszawie, Poland |
| National Bioethics Committee for Medicine and Medical Devices, Bucharest, Romania |
| Ministry of Health National Ethics Committee for Clinical Trial on Medicine, Bucharest, Romania |
| The RF MoHSD, Department of State Regulation of Circulation of Medicines, Ethics Council, Russia |
| Ethics Committee at State Budget Institution of Healthcare (Chelyabinsk Regional Clinical  Oncology Center), Russia |
| Institutional Helsinki Committee Tel Aviv Sourasky Medical Center, Israel |
| Institutional Helsinki Committee Hadassah University Hospital Ein Kerem, Israel |
| Institutional Helsinki Committee The Chaim Sheba Medical Center, Israel |
| Institutional Helsinki Committee Rambam Medical Center, Israel |
| Comite de Etica en Investigacion Instituto Alexander Fleming, Argentina |
| Comissao Nacional de Etica em Pesquisa, Brazil |
| Comite de Etica em Pesquisa da Fundaco PIO XII, Hospital de Cancer de Barretos, Sao Paulo, Brazil |
| Comite de Etica em Pesquisa em Seres Humanos do Hospital Moinhos de Vento, Brazil |
| Comite de Etica em Pesquisa do Hospital Sirio Libanes / Sociedade Beneficente de Senhora, Brazil |
| Comite de Etica em Pesquisa em Seres Humanos Pontificia Universidade Catolica do Parana PUCPR / Centro de Ciencias Biologicas e da Saude /PR Rua, Brazil |
| Comite de Etica em Pesquisa em Seres Humanos do Centro de Pesquisas Oncologicas – CEPON / SC, Florianopolis, Brazil |
| Comite de Etica em Pesauisa da Faculdade de Medicina da USP (CEP-FMUSP), Sao Paulo, Brazil |
| Comite de Etica em Pesquisa da Fundacao Hospital Amaral Carvalho, Brazil |
| Comite de Etica em Pesquisa em Seres Humanos da Fundaco Antonio Prudente Hospital do Cancer AC Camargo/SP, Brazil |
