# Supplementary material for: Eribulin versus dacarbazine in patients with leiomyosarcoma: subgroup analysis from a phase 3, open-label, randomised study
Source: Br J Cancer. 2019 May 8;120(11):1026–32. doi: 10.1038/s41416-019-0462-1 (PMC6738064; doi:10.1038/s41416-019-0462-1)
Supplement: Supplementary file 2 — Supplementary Table 2 [file 41416_2019_462_MOESM2_ESM.docx]

**Supplementary Table 2:** Summary of TEAEs and grade ≥3 TEAEs occurring in ≥ 5% of patients in either arm

| **Category, n (%)** | **Eribulin (n = 156)** | **Dacarbazine (n = 152)** |
| --- | --- | --- |
| Any TEAEs | 154 (99) | 149 (98) |
| Treatment-related TEAEs | 143 (92) | 142 (93) |
| Deaths^a^ | 7 (5) | 3 (2) |
| Other SAEs | 52 (33) | 49 (32) |
| TEAEs leading to study drug dose adjustment |  |  |
| TEAEs leading to study drug withdrawal | 12 (8) | 7 (5) |
| TEAEs leading to dose reduction | 43 (28) | 25 (16) |
| TEAEs leading to study drug interruption | 53 (34) | 51 (34) |
| Grade ≥ 3 TEAEs, ≥ 5% in any group |  |  |
| Any | 108 (69) | 89 (59) |
| Neutropenia | 61 (39) | 24 (16) |
| Leukopenia | 16 (10) | 8 (5) |
| Anemia | 12 (8) | 19 (13) |
| Neutrophil count decreased | 9 (6) | 5 (3) |
| Thrombocytopenia | 1 (1) | 26 (17) |

^a^Grade 5 events in the eribulin arm were large intestine perforation (n = 1), neutropenic sepsis (n = 1), metastases to lung (n = 1), acute respiratory failure (n = 1), pneumonia aspiration (n = 1), and respiratory failure (n = 2). Grade 5 events in the dacarbazine arm were cardiac arrest (n = 1), general physical health deterioration (n = 1), and respiratory failure (n = 1).

SAE, serious adverse event; TEAE, treatment-emergent adverse event.
