# Supplementary material for: Eribulin versus dacarbazine in patients with leiomyosarcoma: subgroup analysis from a phase 3, open-label, randomised study
Source: Br J Cancer. 2019 May 8;120(11):1026–32. doi: 10.1038/s41416-019-0462-1 (PMC6738064; doi:10.1038/s41416-019-0462-1)

**Supplementary Figure 1.** CONSORT diagram of original phase 3 study


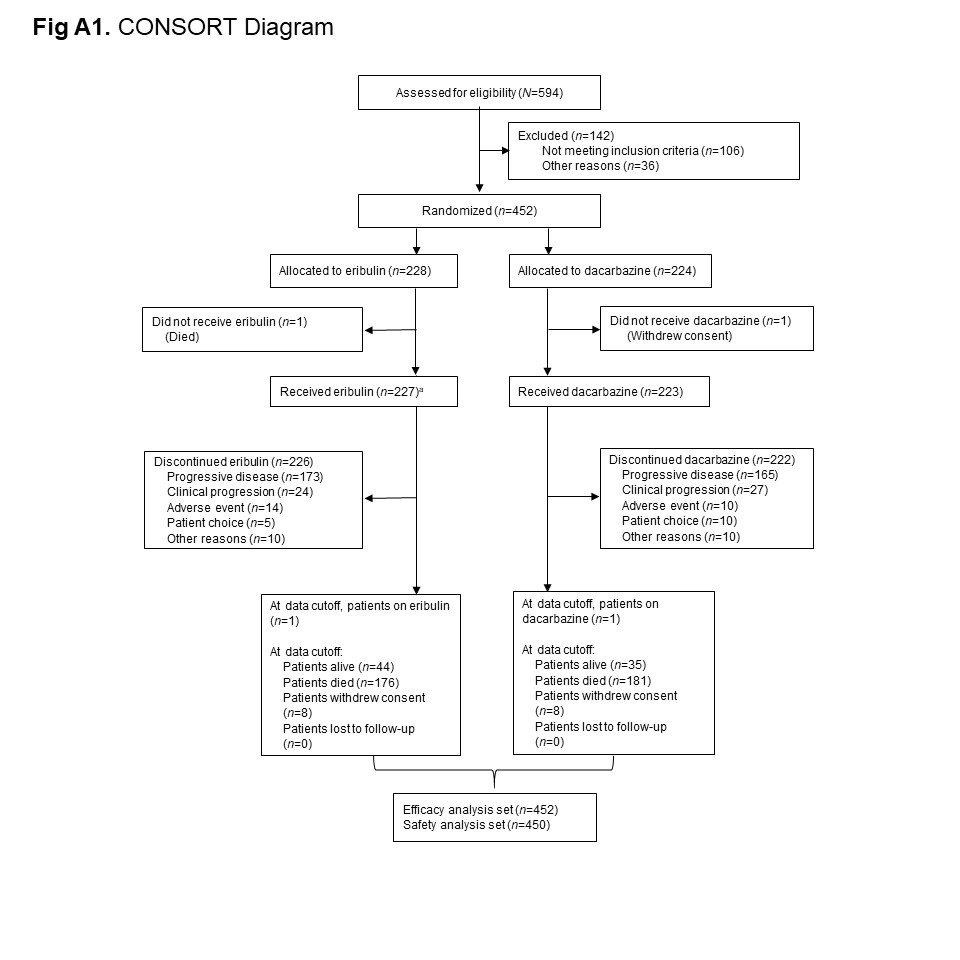


*Includes 1 patient who was assigned to eribulin but treated with dacarbazine.

**Supplementary Figure 2:** Overall survival (A) and progression-free survival (B) by uterine status


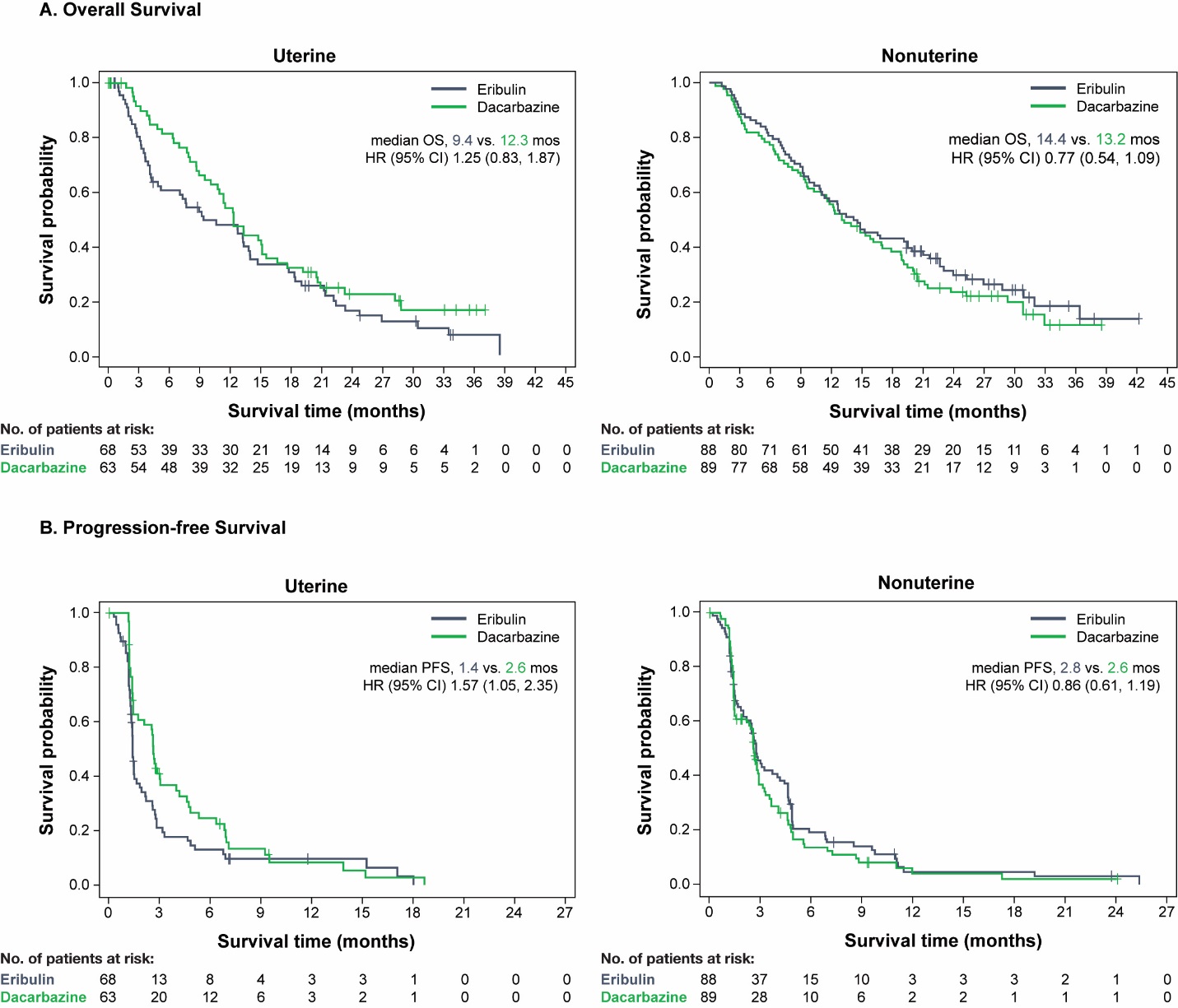


**Supplementary Figure 3:** Overall survival (A) and progression-free survival (B) by gender


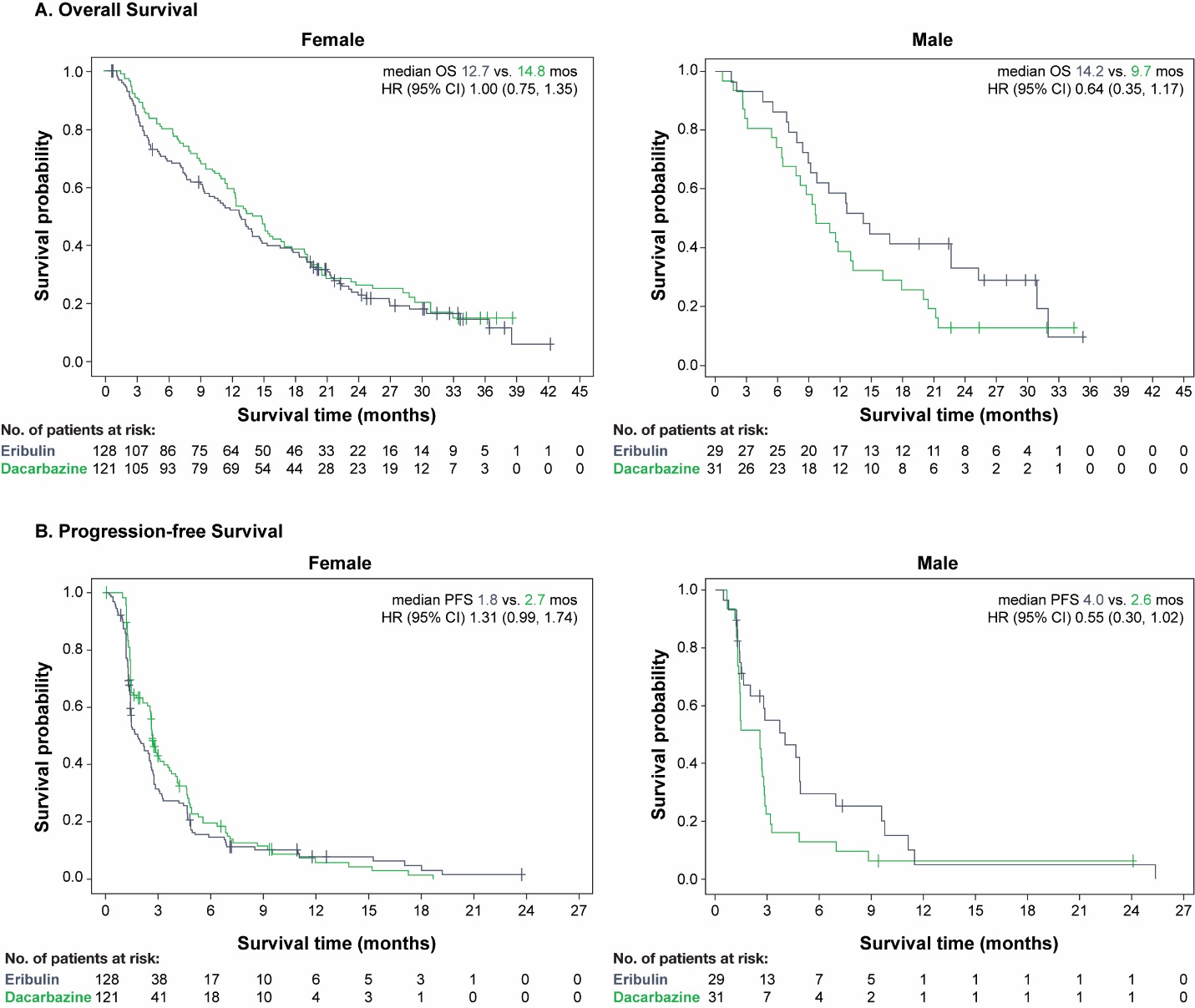

Supplement: Supplementary file 3 — Supplementary Figures [file 41416_2019_462_MOESM3_ESM.docx]
